# Isocyanide Substitution in Acridine Orange Shifts DNA Damage-Mediated Phototoxicity to Permeabilization of the Lysosomal Membrane in Cancer Cells

**DOI:** 10.3390/cancers13225652

**Published:** 2021-11-12

**Authors:** Csaba Bankó, Zsolt László Nagy, Miklós Nagy, Gábor György Szemán-Nagy, István Rebenku, László Imre, Attila Tiba, András Hajdu, János Szöllősi, Sándor Kéki, Zsolt Bacso

**Affiliations:** 1 Department of Biophysics and Cell Biology, Faculty of General Medicine, University of Debrecen, 4032 Debrecen, Hungary; banko@ibiotech.hu (C.B.); rebenku.istvan@med.unideb.hu (I.R.); imre.laszlo@med.unideb.hu (L.I.); szollo@med.unideb.hu (J.S.); 2 Department of Applied Chemistry, Institute of Chemistry, Faculty of Science and Technology, University of Debrecen, 4032 Debrecen, Hungary; sporadicprion@gmail.com (Z.L.N.); nagy.miklos@uni-miskolc.hu (M.N.); keki.sandor@science.unideb.hu (S.K.); 3 Institute of Chemistry, University of Miskolc, 3515 Miskolc, Hungary; 4 Department of Biotechnology and Microbiology, Institute of Biotechnology, Faculty of Science and Technology, University of Debrecen, 4032 Debrecen, Hungary; szeman-nagy.gabor@science.unideb.hu; 5 Department of Computer Graphics and Image Processing, Faculty of Informatics, University of Debrecen, 4032 Debrecen, Hungary; tiba.attila@inf.unideb.hu (A.T.); hajdu.andras@inf.unideb.hu (A.H.); 6 MTA-DE Cell Biology and Signaling Research Group, Faculty of General Medicine, University of Debrecen, 4032 Debrecen, Hungary; 7 Faculty of Pharmacy, University of Debrecen, 4032 Debrecen, Hungary

**Keywords:** phototoxicity, lysosome membrane permeability, DNA damage, QUINESIn method, laser-scanning cytometry (LSC)

## Abstract

**Simple Summary:**

Aside from tissue cell renewal, tumor cells are also produced every day. In ordinary conditions, immunologically controlled cell death mechanisms limit cancer development. There are several cell death processes used for how normal and tumor cells are eliminated at the end of their lifespan. In cancer therapy, cells dying via immunological death are more efficiently eradicated than cells dying by classical apoptosis. Photodynamic treatments with some photosensitizers target lysosomes. Lysosomal death diverts apoptosis to the immunologically more pertinent necrosis-like death pathways. Acridine orange (AO), a well-known photosensitizer, targets lysosomes as well. We have synthesized a new compound abbreviated as DM, a modified AO, and examined details of intracellular processes leading to photodynamic cell death. We have proven that DM targets lysosomes better than AO. Remarkably, with DM, we could visualize an abrupt nuclear DNA release from cells during the photodynamic process. Our work highlights which cellular events may enhance immunological cell death.

**Abstract:**

In cancer therapy, immunogenic cell death eliminates tumor cells more efficiently than conventional apoptosis. During photodynamic therapy (PDT), some photosensitizer (PS) targeting lysosomes divert apoptosis to the immunologically more relevant necrosis-like cell death. Acridine orange (AO) is a PS targeting lysosome. We synthesized a new compound, 3-N,N-dimethylamino-6-isocyanoacridine (DM), a modified AO, aiming to target lysosomes better. To compare DM and AO, we studied optical properties, toxicity, cell internalization, and phototoxicity. In addition, light-mediated effects were monitored by the recently developed QUINESIn method on nuclei, and membrane stability, morphology, and function of lysosomes utilizing fluorescent probes by imaging cytometry in single cells. DM proved to be a better lysosomal marker at 405 nm excitation and lysed lysosomes more efficiently. AO injured DNA and histones more extensively than DM. Remarkably, DM’s optical properties helped visualize shockwaves of nuclear DNA released from cells during the PDT. The asymmetric polar modification of the AO leads to a new compound, DM, which has increased efficacy in targeting and disrupting lysosomes. Suitable AO modification may boost adaptive immune response making PDT more efficient.

## 1. Introduction

Canonical apoptosis occurs during regular tissue replacement as part of the normal physiological homeostasis. As apoptosis does not require any pathological warning signs to signal, it is a silent process. However, infections or cancer cell developments are pathological conditions. These settings must be recognized by immune surveillance, and this is the case, for example, in pyroptosis [1]. Cell death processes rising in pathological conditions may signal danger signals recognized by specific immune cells to the immune system. Then the native or adaptive immune response is initiated against these pathological conditions leading to cell death. This cell death modality is called immunogenic cell death. The minimum requirement for immunogenic cell death is the plasma membrane permeabilization and releasing some intracellular damage-associated molecules (DAMs). ATP, calreticulin, the endoplasmic reticulum protein, HMGB1, the DNA binding nuclear protein, or the cellular RNA and DNA are typical DAMs [2,3,4,5]. Many regulated cell death processes share these primary conditions. For example, in pyroptosis, necroptosis, ferroptosis, or in lysosomal cell death, the plasma membrane gets permeabilized by specific membrane proteins or by oxidative or digestive degradation and opens the route for DAMs. Several harsh physical conditions also lead to the outer surface membrane rupture and the spillover of intracellular contents, such as the necrosis in the accidental cell death.

In the therapy of different cancers, such as skin, breast, or colon, the most established chemotherapeutic agents act in a DNA-damaging manner, leading to tumor cells’ apoptotic death [2,6]. However, activation of the typical apoptosis triggers a rather general anti-inflammatory immunological effect; therefore, killing cancer cells often go unnoticed by the immune system. Phototoxic agents are also effective DNA-damaging agents with the advantage that their action is controllable by light. On the other hand, phototoxicity can target cellular organelles besides DNA, thus acting not only through the destruction of rapidly dividing cells. Damage to extra-nuclear organelles usually causes pyroptosis or necroptosis, which are more immunogenic cell death mechanisms, triggering an adaptive immune response against the tumor cells [3,7,8]. This type of phototoxicity provides an opportunity for the immune system to recognize cancer cells as foreign and eliminate tumors in an autonomous and specific manner. The significance of photodynamic therapy (PDT) comes from the fact that photosensitization causes inflammation, for example, in the skin and can activate the immune system [9,10,11].

**Scheme 1:**
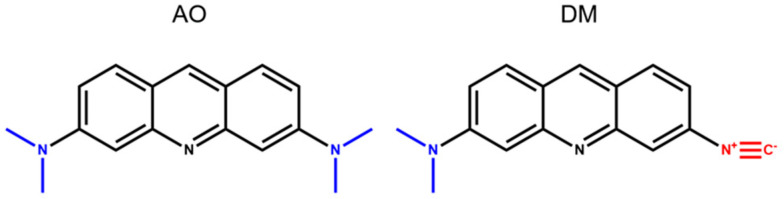
Chemical structure of acridine orange (AO) and 3-N,N-dimethylamino-6-isocyanoacridine (DM, diMICAAc). Molecular weight of AO: 265.35 g/mol, molecular formula: C17H19N3, and chemical name: N,N,N′,N′-tetramethylacridine-3,6-diamine [14]. DM molecular weight: 247.29 g/mol and molecular formula: C_16_H_13_N_3_ [15].

Acridine orange (AO, Scheme 1) is a small molecular photosensitizer (PS) and can serve as a promising agent in PDT [12]. The light-responsive properties and cellular activities of AO are relatively well understood. Although the PS activity of AO has been known for more than 100 years, its use in human PDT has only recently started to spread. The WHO classified AO as non-carcinogenic, and that helped the broadening of the clinical applicability of AO. In several short and long-term clinical trials, both topically and intravenously, AO is toxicologically safe in intact tissues. The medical applications of AO have been mainly in the field of theranostics [13]. Due to its chemical nature, AO accumulates specifically in slightly acidic tumors, and according to its high fluorescence quantum efficiency, it can mark tumor tissues. AO has been successfully used for intraoperative identification of sarcomas or various other neoplasms having intestinal, cervical, or breast origin by precisely identifying tumor resection sites and enhancing tumor cell elimination using the illumination of the resection area during the operation.

AO stains the cytoplasmic and nuclear RNA of living cells green (orthochromatic) but does not bind to live cells’ nuclear DNA. Simultaneously, it colors the nuclear DNA of dying, apoptotic, or necrotizing cells green. AO changes its staining spectrum in fixed cells. In dying or fixed cells, AO binds to RNA as dimers (stacking two molecules), staining it intensive red (metachromatic). AO intercalates to the DNA of fixed cells as monomers and stains denatured DNA green [16,17]. Another interesting phenomenon suitable for studying lysosomal membrane integrity in live cells is the AO-sensitized photodestruction of lysosomes. In living cells with adequate metabolic activity, besides staining RNA green, AO accumulates in lysosomes. Concentrated AO in lysosomes also forms dimers, causing the characteristic metachromatic shift of the emission spectrum and stains lysosomal vesicles red. When cells are exposed to blue light, AO-generated radicals damage the lysosomal membrane; red vesicles turn to green firework-like flashes by exploding spectacularly as concentrated AO dilutes into the cytosol [16,18,19,20]. Proliferating tumor cells, according to the Warburg effect, are more acidic than normal tissue. Acidification explains the spontaneous accumulation of AO in neoplasms [21]. Other tumor-related variations are increasing RNA and DNA content [13] or multiplying intracellular membrane systems, such as the frequent expansion of lysosomes, all of which further strengthen the accumulation of AO in tumors. The overexpression of the vesicular autophagosome-lysosome system is particularly characteristic of tumors having multidrug resistance. In these tumors, chemotherapeutic agents primarily accumulate and are metabolized in lysosomes. A lysosomal exocytosis is a handy option for eliminating chemotherapeutic agents, representing an additional degree of freedom in tumor evolution. The AO can selectively recognize and attack the most resistant types of tumors through the PDT, making its research precious. To compare the action of AO with that of very closely related compounds would reveal even more in-depth insights into the cell biological mechanism of action of these photodynamic compounds and could help their use in clinics.

Recently, we reported the synthesis and characterization of a novel fluorescent dye family called amino-isocyanoacridines for pH sensing in the physiological range and live-cell imaging [15]. These acridine derivatives showed altered properties compared to AO, such as reduced basicity, enhanced solvatochromic emission range, low quantum yield in water, and easy tuning of their fluorescent behavior by complexation with metal ions. It was also shown that among these novel derivatives, the 3-N,N-dimethylamino-6-isocyanoacridine (DM) has the most favorable properties in terms of excitation wavelength, fluorescent emission range, and preserving the original cell morphology. AO, compared to DM, being a symmetric molecule, can be considered almost nonpolar, especially in the ground state. In contrast, the substitution of one of the electron-donating (D) dimethylamino groups of AO (Scheme 1) to an electron-withdrawing (A) isocyanide group enhances the polarity of the DM molecule considerably (the outwardly oriented, negatively charged end of the DM is the isocyano, while the positive is the dimethylamino group). The basis of the high polarity is the internal charge transfer (ICT) between the two different groups (D-A) through the aromatic acridine ring, which results in an enhanced dipole moment change (∆µ = µ_e_ − µ_g_) between the excited and ground state (∆µ = 2 and ∆µ = 8 calculated in debye for AO and DM, respectively). The more considerable dipole moment change in DM is the reason for its enhanced solvatochromic range compared to that of AO and its potential application as pH probes for lysosomes at physiologic cellular conditions [15]. Thus, we wondered whether DM could be an efficient lysosomal probe of human cells.

Acridine derivatives often bind to nucleic acids, thus frequently damaging the genetic material in PDT. This process, unfortunately, may lead to immunologically silent apoptosis. However, lysosomes serve as the extra-nuclear target of acridine derivatives, and their disruption directs the mechanism of cellular death towards the immunologically effective necrotic-like cell death. Therefore, it was interesting to compare the DNA-damaging and lysosome-destructive effects of AO quantitatively to that of our new DM compound.

To learn more about the mechanism of PDT action, we evaluated the cell biological properties of the AO and DM in detail. We ascertained that both AO and DM have comparable actions with slight alterations in some scenarios. AO affected the DNA more than DM, giving more extensive apoptosis-like DNA damage on the molecular level. The DM, however, caused more lysosomal injury and resulted in more massive and abrupt necrosis-directed cell damage. This behavior is rooted in their chemical structure; the DM has more significant polarity than AO, explaining its pronounced lysosomotropism and sharper imaging of lysosomes. Moreover, using this novel dye during the photodynamic cell treatment, the real-time release of damaged DNA from cell nuclei as shockwaves of DNA fragments could be visualized, as far as we know, for the first time.

## 2. Materials and Methods

### 2.1. Chemicals and Cells

3-N,N-dimethylamino-6-isocyanoacridine (DM) was obtained in a two-step reaction starting from 3,6-diaminoacridine. In the first step, one of the amino groups of 3,6-diaminoacridine was converted to isocyanide using dichlorocarbene formed in situ from chloroform in a basic medium. The product of the first step is 3-amino-6-isocyanoacridine (ICAAc). During the second step, the methylation of the free amino group of ICAAc was carried out with CH3I/KOH in toluene. The pure dimethylamino derivative (DM) was obtained after purification by column chromatography. The detailed synthetic procedures and characterization of DM and the intermediates are found in the Supporting Information of [15]. All chemicals were from (Sigma Aldrich, Budapest, Hungary) unless otherwise stated.

The HeLa (#CRM-CCL-2, ATCC) human cervix epithelial adenocarcinoma cell line and the SK-BR-3 (#HTB-30, ATCC) human breast cancer cell line, here labeled as SKBR-3, was obtained from the American Type Culture Collection (Rockville, MD, USA). The OCM-1 human melanoma cell line was kindly provided by Dr. H. M. H. Hurks, Department of Ophthalmology, Leiden University Medical Center, Leiden, The Netherlands [22]. The 3T3-MDR1 cell line is the human ABCB1 protein transfected NIH 3T3 mouse fibroblast cell line expressing a high level of the human transporter (named originally as NIH 3T3 MDR1 G185 cells) is a kind gift from Michael Gottesman (National Institutes of Health, Bethesda, MD [23]. OCM-1 cells were maintained in RPMI, while other cell lines were maintained in DMEM (Livetech, Raleigh, NC, USA). Both media were supplemented with 10% fetal calf serum (Thermo Fischer Scientific, Budapest, Hungary), 2 mM glutamine, PS (10 U/mL penicillin, and 10 µg/mL streptomycin) or 10 µg/mL gentamycin, and with Phenol-Red (Livetech) in a humidified atmosphere with 5% CO_2_ at 37 °C. 

### 2.2. Cellular Toxicity Measurements

For cellular toxicity measurements, 10,000 cells were seeded in each well in a Corning Costar 96 well plate. Dilution series of 9.4 × 10^−5^, 3.8 × 10^−5^, 1.9 × 10^−5^, 9.4 × 10^−6^, 4.7 × 10^−6^, 2.4 × 10^−6^, 1.2 × 10^−6^, 5.9 × 10^−7^, 2.9 × 10^−7^, 1.5 × 10^−7^ M from AO and 1.0 × 10^−4^, 4.0 × 10^−5^, 2.0 × 10^−5^, 1.0 × 10^−5^, 5.1 × 10^−6^, 2.5 × 10^−6^, 1.3 × 10^−6^, 6.3 × 10^−7^, 3.2 × 10^−7^, 1.6 × 10^−7^ M from DM were applied in four parallel experiments. After the 24 h incubation time, 100 µL MTT solution (0.5 mg/mL in PBS: 150 mM NaCl, 3.3 mM KCl, 8.6 mM Na_2_HPO_4_, and 1.69 mM K_2_HPO_4_, pH 7.4) was added to each well. The plates were incubated for 2 h at 37 °C, then the wells were aspirated, and the MTT formazan was extracted with 200 µL of DMSO aided by gentle agitation on a shaker. After 30 min at room temperature, a Synergy HT multi-detection microplate reader (BioTek, Winooski, VT, USA) recorded the absorbance values at 565 and 680 nm. The instrument was blanked beforehand on a row not containing cells, and the absorbance was corrected by subtracting absorbance values measured at 680 nm from the corresponding absorbance of 565 nm. Percentage viability (respiratory competence) of the population of cells in each well was expressed as:

(Corrected absorbance of treated cells/corrected absorbance of untreated cells) × 100.

### 2.3. Spectrofluorimetry Measurements

The excitation and emission spectra were recorded in a quartz cuvette of 1.0 cm optical length using a Jasco FP-8200 fluorescence spectrophotometer (JASCO Corporation, Tokyo, Japan), equipped with a Xe lamp light source. Data were recorded at 20 °C, using 2.5 nm excitation, 5.0 nm emission bandwidths, and 200 nm/min scanning speed at “normal” sensitivity. First, we recorded all spectra of solutions containing one million HeLa cells/mL labeled with 2.4 × 10^−6^ M AO or 2.5 × 10^−6^ M DM. Then, in control experiments, these parameters were recorded with the same settings but without the cells.

### 2.4. Confocal Microscopy and Spectral Imaging

Cells were seeded in ibidi slide (8-well ibidi µ-Slide, Zenon Bio Kft, Szeged, Hungary) at a concentration of 30,000 cells/well and the next day labeled with 2.5 × 10^−6^ M AO and DM for 2 h. Then, fluorescence and bright field 16-bit images were taken by a Zeiss LSM 880 confocal microscope (Carl Zeiss microscopy Gmbh, Oberkochen, Germany) using a ×40 C-Apochromat water immersion objective applying 405, 488, and 543 nm laser excitation and blue (410–474 nm), green (499–540 nm), and red (554–629 nm) emission channels. For spectral imaging, the same wavelengths excitation laser lines were applied, and total emission ranges at the three excitations were 410–695, 499–695, and 553–695 nm, respectively. A fair number of images were made at every 18-nm span with a 32-channel linear detector system in these broader ranges.

### 2.5. Investigation of Lysosomal Morphology Applying LysoOrange

On the previous day of the measurement, 30,000 HeLa cells were seeded to each well of an eight-well ibidi slide. In order to visualize lysosomes, we stained the cells with a sub-vital lysosomal probe in a humidified atmosphere with 5% CO_2_ at 37 °C. 20 µL LysoOrange (ab176827 CytoPainter, Abcam, Cambridge, UK) was added to 10 mL RPMI medium, and from this lysosome labeling solution, 300 µL was used to an ibidi well. After 15 min of incubation, images of cells were recorded at 543 nm excitation wavelength. Testing the effect of PS and light on lysosomal morphology, one plate was treated with 2.5 × 10^−6^ M AO and another with the same concentration of DM. The incubation time was one hour under the same cell culture conditions. After the incubation with PS, one cell was selected, and an image was taken (before irradiation). Then, with 50-s continuous imaging, 1000 frames were recorded (illumination), and a 20 frame per second (fps) video was constructed from these images of the same cell. After the illumination, the medium with the PS was replaced by the lysosome labeling solution. After the lysosomal staining at 37 °C, another image was taken from the same cell. The procedure was repeated both for the AO and DM.

### 2.6. Phototoxicity Measurements with Blue LED Lamp Applying MTT Bioassay

Cells were seeded at 10,000 cell/well concentrations one day before the experiment. To test the phototoxicity of the compounds, we set up the following system. A 470 nm wavelength LED lamp (Alustar 900808; Ledxon, Geisenhausen, Germany) was placed 10 cm above and oriented towards the sample in a 96-well plate situated in a conventional cell culture incubator. The optical axis of the illumination was centered to the center of the flat-bottom plate. For the light treatment, the attached cells in different plates were irradiated for increasing times. Cell viability was determined by applying the MTT assay, as described above. The blue LED lamp had a 30-degree cone with a 23.3 lm luminous flux, which, according to our calculations, corresponded to a 4.45 mW/cm^2^ fluence rate at the sample level.

### 2.7. Laser-Scanning Cytometry (LSC) and Live-Cell Imaging

Laser-scanning cytometry was used for fluorescent live-cell imaging with parallel fluorescent detection of cell death events and video recording; QUINESIn measurements were utilized to evaluate DNA superhelicity, histone quantities, and DNA integrity in single cells [18]. The slide-based laser-scanning iCys Research Imaging system (Thorlabs Imaging Systems, Sterling, VA, USA) was equipped with four lasers, two photodiodes for chromatic absorbance detection, and four photomultipliers for fluorescence detection, and with an ibidi microscope-stage incubator (Zenon Bio Kft, Szeged, Hungary). Imaging of the samples was set up in one or several predefined regions of interest (ROI), where scanning took place point by point, either at once or separately following one another by the multiple laser lines. A fixed offset from the bottom of the well was applied during scans focusing on the middle plane of cells. User-defined areas in the specimen with optimal cell density were marked as regions of interest and scanned in an automated manner.

For live-cell imaging and video recording, cells were kept at 37 °C with 5% CO_2_ air condition and 90% humidity. Cells were seeded at 30.000 cells/well into ibidi slides 24 h before the measurement, and cells were treated with 2.5 × 10^−6^ M AO or DM. Propidium iodide was added to each sample at 7.5 × 10^−6^ M final concentration. One field image was recorded at the region of interest (ROI), with a 500 × 184 μm^2^ area. The field image was 1000 × 768 pixels; hence the size of one pixel was 0.5 × 0.24 μm. One laser-scanning illumination cycle lasted for 2.9 s, and illuminations were replicated every 5 min. Imaging was repeated in 63 consecutive cycles. The arising fluorescence signals of the 405, 488, 561, and 633 nm lasers were collected by a 40∙LWD (NA 0.6) objective into four detection channels (wavelengths are given as mean/range in nm): blue (480/40 nm), green (530/30 nm), red (580/30 nm), and long-red (675/50 nm). In some live-cell experiments, just the 488 nm laser and the green and far-red channels were used. The measured laser power of the 488-laser line was 0.30 mW ± 0.05 (mean ± SD), which provided a 0.32 W/cm^2^ fluence rate at the sample. After the LSC scan, wide-field fluorescence images were taken by an Olympus DP71 color camera (Olympus Hungary Kft., Budapest, Hungary) applying typical violet/blue, blue/green, and green/red filter cubes with mercury vapor lamp illumination.

### 2.8. Illuminated QUINESin Method

We described earlier the original QUINESin method [24]. In this work, we slightly modified this method to verify the effect of light illumination for histone elution (Figure 6a).

In the beginning, live cells were embedded into low melting point agarose. Before embedding, the wells of ibidi slides were coated with 1% (*m*/*v*) low melting point agarose. A total of 150 μL liquid agarose, diluted in distilled water, was dispensed into each well and was immediately removed so that a thin agarose layer remained on the surfaces and was left to solidify on ice for 2 min. Then, slides were kept at 37 °C until the surface of the wells dried out. This coating procedure was repeated once more on the same chambers. Embedding was performed, keeping cells and agarose at 37 °C. The cell suspension containing 6 × 10^6^ cells/mL was mixed with 1% low melting point agarose diluted in PBS at a v/v ratio of 1:3. 22 μL of the cell agarose suspension was dispensed in the middle of the wells and the chambers were covered with homemade rectangular plastic coverslips cut out from a 200 μm thick, medium weight polyvinyl chloride binding cover (Fellowes, Inc., Itasca, IL, USA). The cells were left to sediment on the coated surface of wells for 4 min at 37 °C and then kept on ice for 2 min. When the agarose solidified, a 300 μL ice-cold complete culture medium was added to each well, a step aiding the removal of coverslips.

#### 2.8.1. Illumination of Agarose Embedded Live Cells with LSC for the Illuminated QUINESin Assay

After live-cell embedding, an area of one field image was irradiated by the 488 nm laser light in each well by making images with the LSC system (Figure 6a). The illumination of the exact location and the imaging was repeated in subsequent consecutive cycles, as described above in the LSC section.

#### 2.8.2. Preparation of Nuclei/Permeabilization and Histone Eviction by Salt or Intercalators

The agarose-embedded cells at the bottom of the wells were washed with 500 μL ice-cold PBS three times for three minutes, then permeabilized with 500 μL ice-cold 1% (v/v) Triton X-100 dissolved in PBS/EDTA (5 mM EDTA in PBS) for 10 min. This step was repeated once more. After permeabilization, the nuclei were washed by a regular three-minute in-well bath with 500 μL ice-cold PBS/EDTA three times and were treated with different NaCl solution concentrations on ice. The nuclei were washed with 500 μL of ice-cold salt solution for 60 min. After this step, the nuclei were bathed in-well with 500 μL of ice-cold PBS/EDTA three times. NaCl was diluted in PBS/EDTA; the salt concentrations indicated on the X-axes of the graphs in all panels of Figure 6b show the total NaCl concentrations together with NaCl present in the PBS buffer. Analyses of the curves were performed by SigmaPlot 12.0, using either ‘Sigmoid 3 parameter’ (in the case of linear plots) or ‘Standard curves: Four Parameter Logistic Curve’ (in the case of logarithmic plots) curve-fitting subroutines. The number of analyzed G1 nuclei was between 200 and 1000 cells/well, out of the about 500–2000 cells scanned. All SEM values in Figure 6b were calculated from the cell population’s data points analyzed in the given experiment. 

#### 2.8.3. Immunofluorescence Labeling

After salt treatment, the samples were incubated with 500 μL 5% (*m*/*v*) Blotto Non-Fat Dry Milk (Santa Cruz Biotechnology Inc., Santa Cruz, CA, USA) PBS/EDTA for 30 min on ice to decrease nonspecific binding of the antibodies. The blocking solution was washed out by an in-well bath with 500 μL ice-cold PBS/EDTA three times. Indirect immunofluorescence labeling was performed using rabbit polyclonal anti-H2A (Abcam) primary antibody, diluted in 150 μL of PBS/EDTA/1% BSA (PBS/EDTA supplemented with 1% *w*/*v* bovine serum albumin), at 4 °C, overnight at a titer of 1:800. After labeling with the primary antibody, the nuclei were washed with 500 μL ice-cold PBS/EDTA three times for 10 min. Labeling with the secondary antibody was performed in 150 μL PBS/EDTA for two hours on the ice, using Alexa fluor 647 conjugated goat anti-mouse IgG or goat anti-rabbit IgG antibodies (Thermo Fisher Scientific, Budapest, Hungary). The secondary antibody was also used at a titer of 1:800, diluted in PBS/EDTA from 2 mg/mL stock solutions. After labeling with the secondary antibody, the agarose-embedded nuclei were washed with 500 μL ice-cold PBS/EDTA three times for 10 min. The samples were then fixed in 1% formaldehyde (dissolved in PBS/EDTA) at 4 °C, overnight. After fixation, the embedded nuclei were in-well washed with 500 μL ice-cold PBS/EDTA three times and stained with 200 μL 5 μg/mL propidium iodide (dissolved in PBS/EDTA) for 30 min on the ice. The stained nuclei were incubated in the well with 500 μL ice-cold PBS/EDTA three times. Fluorescence intensity distributions were recorded using the LSC imaging system, as described above.

### 2.9. Quantification of Lysosomes and Lysosomal Membrane Integrity

One day before the measurements, 10,000 HeLa cells were seeded to each well of 96-well plates. The dilution series of 3.8 × 10^−5^, 1.9 × 10^−5^, 9.4 × 10^−6^, 4.7 × 10^−6^, 2.4 × 10^−6^, 1.2 × 10^−6^, 5.9 × 10^−7^, 2.9 × 10^−7^, 1.5 × 10^−7^ M from AO and 4.0 × 10^−5^, 2.0 × 10^−5^, 1.0 × 10^−5^, 5.1 × 10^−6^, 2.5 × 10^−6^, 1.3 × 10^−6^, 6.3 × 10^−7^, 3.2 × 10^−7^, 1.6 × 10^−7^ M from DM was applied in four parallel plates. As an untreated control, glucose-PBS (PBS supplemented with 8 mM glucose) was added to cells; the blank contained only glucose-PBS without cells. As for positive controls, cells were treated with LLoMe (L-leucyl-L-leucine methyl ester, Sigma Aldrich, in 25 mM), a known lysosomal damaging agent. After 45 min of incubation in a humidified atmosphere with 5% CO_2_ at 37 °C, the fluorescence intensity of the wells was measured by the Synergy HT multi-detection microplate reader. The excitation filter was 485/20 in both cases, while the emission filter was 528/20 for AO and 590/20 for DM. The fluorescence intensity parameters in the presence of photosensitizers were measured 10 times in each well in order to damage lysosomal membranes. Then solutions were removed, and the cells were stained with LysoOrange suspension similar to confocal microscopy. Wide-field fluorescence micrographs were finally taken from each well with the LSC imaging system color camera using green/red filter cubes and 20× objective described above.

### 2.10. Image Analysis of Cellular Lysosome Content

We have determined the lysosome content of cells of camera images for every well. The lysosome content of cells is determined in the images of wells as a quotient of the sum of pixels in every lysosomal region and all cells’ pixels. This method gives an approximation for the lysosome-cell ratio in an image. A detailed description of the image analysis is provided in the supplementary materials.

### 2.11. Movies

Images were captured at defined intervals by imaging cytometry or confocal microscopy, then processed with the software of the systems and used to make original video frames. After further image enhancements, we have produced final movies played at a rate of 7 frames per second (fps) using the Fiji program.

### 2.12. Statistical Calculations

For data comparison, we have applied the Student’s *t*-test, Tukey’s multiple comparisons test, nonlinear regression dose-response inhibition semi-logarithmic normalized curve fitting, and comparison of fits statistical analysis using the GraphPad Prism 8.4.3 for Windows software.

**Figure 1:**
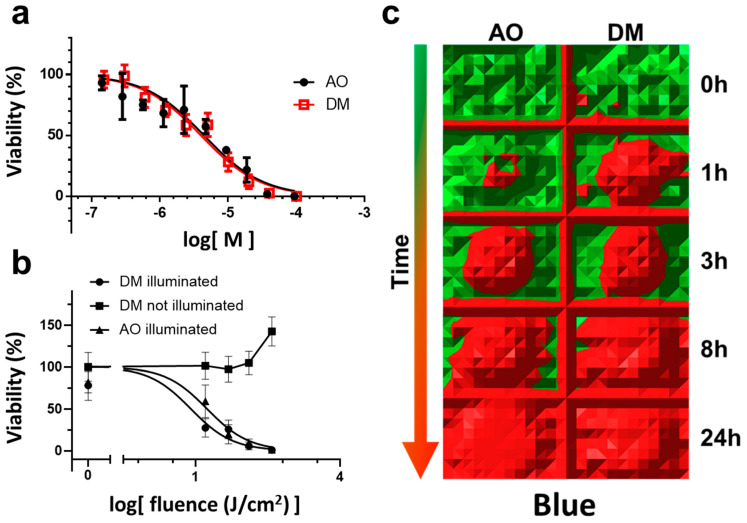
Viability of HeLa cells labeled with AO or DM. (**a**) AO and DM have nearly identical toxicity in HeLa cells. The 24-h toxicity of the two dyes was determined with an MTT assay. The LD_50_ toxicity values were 4.4 × 10^−6^ M for AO and 4.0 × 10^−6^ M for DM (the difference is not significant). (**b**) DM has higher phototoxicity than the AO in a continuously illuminated LED lamp experiment. Half of the cells were killed after 7.9 and 17.3 J/cm^2^ light fluences in 2.5 µM DM and AO sensitized cells. Phototoxicity was evaluated using a 24-h MTT assay in HeLa cells. The data shown in (**a**,**b**) are mean values with error bars representing the standard error of the mean. (**c**) Pseudo-color images of the 96-well plates of the representative raw data of the MTT assay-based experiments of the phototoxicity kinetics arranged on time. The red color shows the ratio of dead cells according to MTT values at the location of wells, while live cells are denoted by green. The left five plates show AO, and the right ones show DM; from top to bottom, the corresponding incubation times are 0, 1, 3, 8, and 24 h.

In detail, for the AO and DM toxicity measurements in the dark, four experiments were conducted (Section 3.2, Figure 1a). A representative with four parallels for the selected drug concentrations was evaluated in GraphPad. The nonlinear regression dose-response inhibition semi-logarithmic normalized curve fitting was performed. The statistical evaluation of the comparison of fits did not show a significant difference.

For continuous LED light phototoxicity experiments, a representative of three experiments was selected and presented in Figure 1b,c (Section 3.3). Here an entire 96-well plate with 2.5 µM drug treatment (AO and DM) was used for one time-point of the phototoxic kinetic measurement (Figure 1c) applying the MTT viability assay. At one time-point, the MTT values of the middle 36 wells (the center of all 96-well plates was centered to the optical axis of the LED) of the entire plate were first normalized to the average MTT value of one full unilluminated plate of 96 wells. Then the normalized 36 values were averaged. The mean and SEM of these normalized data were plotted (y viability value) as one fluence value in Figure 1b. The irradiation value (x fluence value of Figure 1b) was calculated as a 10-base logarithm of the product of the time of illumination in seconds and the fluence rate (mW/cm^2^) of the illumination (calculation details in Section 2.6). Then, DM and AO normalized curves were calculated and fitted according to the log(dose)-response inhibition model, and IC50 values were statistically compared using the F test for sum-of-squares in GraphPad. The *p*-value was less than 0.0001.

Five experiments were conducted for LSC phototoxic experiments (Section 3.6, Section 3.7 and Section 3.8) and two videos constructed with Fiji. One video was selected for quantification. All image fields contained approximately 70–150 dye-loaded cells. Dye content representing intensity profiles was measured on whole images (Figure 5c,d) or in the selected ten-pixel diameter circular region of interest (Figure 5g,h).

For QUINESIn experiments (Section 3.10), we have selected one representative example out of the three similar experiments for Figure 6. On plots of Figure 6b, every data point was calculated from 50–500 individual cell nuclei. 

For lysosomal photodamage experiments, plate-reader experiments were repeated three times. One plate was chosen, and images were recorded for all of the 96-wells. These images were processed for further image analyses, as detailed in the supplementary materials. For statistical analysis of data in Figure 8b, AO and DM samples were compared by *t*-test. Tukey’s multiple comparisons test was performed for all the samples. GraphPad’s curve fitting was performed by nonlinear regression using the log(inhibitor) dose vs. normalized response model. The fitted curves least-square data were tested using the F test (Figure 8c).

## 3. Results and Discussion

### 3.1. Fluorescence Absorption and Emission Properties of the Photosensitizers

To clarify how the substitution of the dimethylamino group to isocyano moiety affects the steady-state photophysical properties of compounds to be used in cellular applications, we have compared both the light absorption and fluorescence light emission spectra of the two dyes in an aqueous medium (PBS pH 7.4) in the absence and the presence of HeLa cells (Figure S1). Without cells, DM has a broader and relatively flat absorption spectrum in the 400–500 nm range, while AO has a sharp and twice as high absorption maximum, close to the 488 nm laser line. Cells did not alter peak positions on the absorption spectra of dyes; however, a light scattering of cells significantly increased the absorbance values, especially towards the shorter wavelength (Figure S1, upper panels). Remarkably, when cells were excited at 480 nm, AO had approximately a twenty-times higher fluorescence intensity, at around 530 nm, than DM. As referenced to this peak, the emission maximum of DM is red-shifted at about 45 nm. The presence of cells did not significantly alter the emission spectra of dyes (Figure S1, lower panels).

### 3.2. DM and AO Has Comparable Toxicity in Dark

Next, we wondered whether DM’s cellular toxicity is different from that of AO in a 24-h MTT assay. The LD_50_ toxicity value in human HeLa cells for both dyes was approximately 4 µM (Figure 1a), with about a 10% difference (4.4 × 10^−6^ M for AO and 4.0 × 10^−6^ M for DM. The 95% confidence levels for AO was: 3.4 × 10^−6^ to 5.8 × 10^−6^ M, and for DM: 3.3 × 10^−6^ to 4.8 × 10^−6^ M).

### 3.3. DM Is More Phototoxic Than AO in Continuous Blue Light Illumination

AO has been known for its photosensitizing property; thus, we were curious whether this property is altered by DM. We illuminated HeLa cells treated with photosensitizers using a 470-nm LED light having a wavelength close to the absorption maxima of the dyes for increasing periods (0, 1, 3, 8, and 24 h). Then we examined the viability of cells in a 24-h MTT assay. Half of the cells sensitized by applying 2.5 µM DM or AO were killed after 30 or 65 min of continuous light exposure, so DM initiated HeLa cell death earlier than AO (Figure 1b,c). The LD50 fluence of the blue light illumination was significantly different for the two photosensitizers: 7.97 J/cm^2^ for DM and 17.30 J/cm^2^ for AO. The 95% confidence levels were 6.4 and 9.7 J/cm^2^ for DM, and 15.0 and 19.9 J/cm^2^ for AO (Figure 1b), *p* = 0.0001.

### 3.4. The Optical Properties of Cells Stained by DM or AO; DM Marks Lysosomes Better at the 405-nm Laser Line Than AO

**Figure 2:**
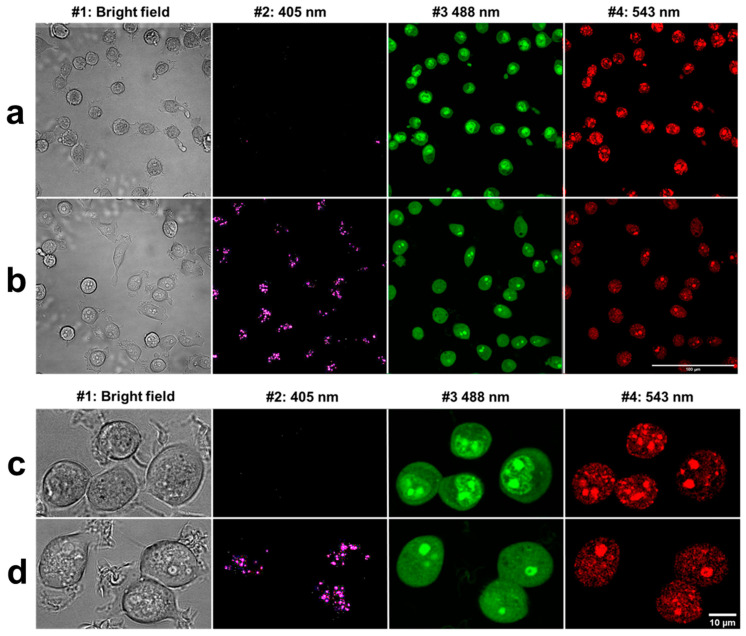
Confocal fluorescence microscopic images of HeLa cells labeled with AO or DM. Overview (**a**,**b**) and high-resolution images (**c**,**d**) of HeLa cells after AO (**a**,**c**) or DM (**b**,**d**) staining. Different excitation and emission settings were applied for the two dyes to optimize images for the intensity conditions of the captured image. From left to right: bright field of view illumination, 405, 488, and 543 nm laser light excitation for blue, green, and red emissions. One set of representative color images out of three similar experiments are displayed in this figure. The size marker corresponds to 100 µm for images (**a**,**b**) and 10 µm for images (**c**,**d**).

It is known that AO is a suitable lysosomal probe in red fluorescence emission with green light excitation [17]. Consequently, we were interested in the optical properties of the new compound as well. We have performed several microscopic examinations and concluded that the two dyes stain human cells comparably well at the same 1–3 µM concentration range in short-term labeling (in culture medium for 15–60 min at 37 °C). Confocal examinations demonstrated that cells do not indicate any morphological signs of toxicity in these short-term applications (3–4 h in physiological conditions, Figure 2, bright field images).

However, DM indicated better discrimination of the lysosomal type vesicles in the cytoplasm of the HeLa cells at 405 nm laser light excitation and the blue fluorescence emission range than AO did at 543 nm excitation and in the red emission range (Figure 2, 405 and 543 nm images). At 488 nm excitation and green fluorescence emission, AO had much brighter fluorescence (Figure 2, 488 nm images) than DM. At this 488 nm wavelength, both dyes marked the nucleoli of live cells the best, probably the ribosomal RNA. Interestingly, contrary to AO, DM did not discriminate nuclear DNA from other cytoplasmic regions of cells. AO-stained DNA with a continuously intensifying manner, which probably parallels how illuminating light stressed the cells increasingly. This differential DNA staining is probably rooted in the different fluorescence quantum efficiency of the two dyes. At higher excitation and emission regimes, neither AO nor DM could discriminate either lysosomes or nucleoli because both dyes marked all components to a similar level (Figure 2, 543 nm images).

### 3.5. The Human ABCB1 Transports DM Less Than AO

**Figure 3:**
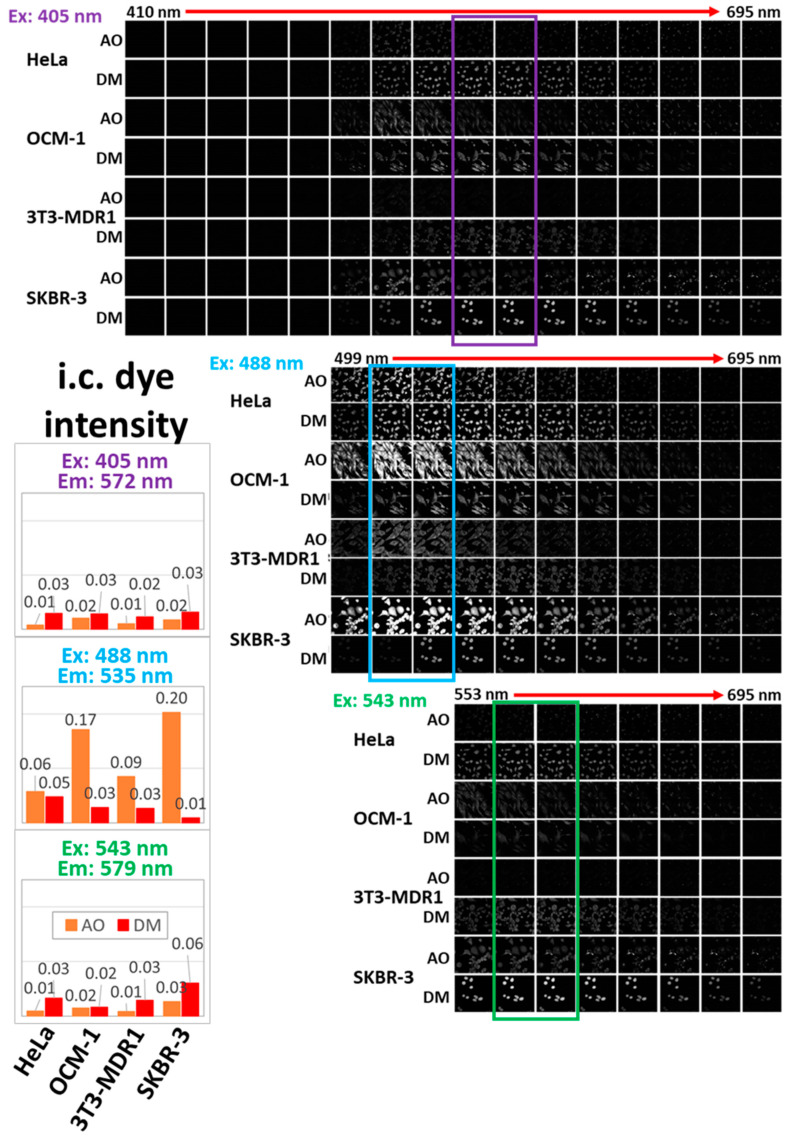
Spectral imaging of fluorescently labeled HeLa, OCM-1, 3T3-MDR1, and SKBR-3 cells. Differences in UV-Vis absorption and fluorescence emission between the two dyes in the presence of cells are illustrated using spectral imaging. Laser excitations at 405, 488, or 543 nm were used to capture images of the three panels from top to bottom. The fluorescence emission spectrum of the three panels from top to bottom contained 16, 11, or 8 pieces of 18-nm bands spanning the emission ranges of 410 to 695, 499 to 695, or 543 to 695 nm. The bar chart shows the relative intracellular dye intensities of the AO and DM at their maximum emission intensities (572 ± 18, 535 ± 18, 579 ± 18 nm) according to the spectral imaging at the three different laser excitations (405, 488, 543 nm), respectively. The intensities were determined using the Gel plugin of the Fiji software by measuring the total area under the curve of the cell intensity profile of images. One representative image series of three similar experiments is displayed in this figure.

We performed confocal spectral imaging with two additional human and one mouse cell line (SK-BR-3, OCM-1, 3T3-MDR1) to see whether other cells having different tissue and species origin demonstrate staining patterns similar to or different from that of the human HeLa cells. Spectral imaging supported that all human and mouse cell types were stained with comparable spectral patterns, and staining did not depend profoundly on the origin of the cells (Figure 3). However, the overall intensity of the cell staining was different. Under the same excitation and detection conditions, image intensities identify the 490 nm excitation maximum and the higher fluorescence quantum efficiency of the AO correctly and the broader excitation and emission ranges of the DM. AO stained cells in the following descending order: SKBR-3 > OCM-1 > HeLa > 3T3-MDR1. Compared to AO, DM was taken up in different order: SKBR-3 > HeLa > 3T3-MDR1 > OCM-1. Intensity deviations support the idea that various membrane carriers and transporters are involved in dye transportation. 3T3-MDR1 mouse cells were derived by stable transfection of NIH-3T3 cells using the human MDR1 multidrug transporter gene. AO is a known MDR1 transporter substrate, which explains the minimal AO uptake by the 3T3-MDR1 cells. However, the DM transport efficiency of the MDR1 transporter (and it is probably valid for other involved transporters) is different from that of AO, which is why the dye uptake order of the cell lines is different for AO and DM. These cells showed a greater intensity with DM, suggesting that DM is a worse substrate of the human ABCB1 membrane transporter than AO. This property of DM is beneficial for lysosomal staining.

### 3.6. Governing Phototoxicity in Single Cells by Imaging Cytometry

Acute cell death events, when cell membrane ruptures, can be visualized; therefore, we decided to monitor the phototoxic process. We set up a cell follow-up experiment applying an iCys laser-scanning cytometry imaging system. HeLa cells were seeded into ibidi slides and imaged in culture conditions utilizing a stage incubator. Cells were visualized by scanning one field of view, which took 2.9 s in one cycle. Cycles were repeated every 5 min 63 times, giving approximately 5 h of cell monitoring and 180 s of total laser light exposure in a fractionated manner. Since we measured fluence at the sample level, we could conveniently control the light illumination energy to cells by counting the number of applied cycles. To detect membrane rupture at the death of cells, we supplemented the medium containing photosensitizers with a membrane-impermeable red DNA dye, propidium iodide.

**Figure 4:**
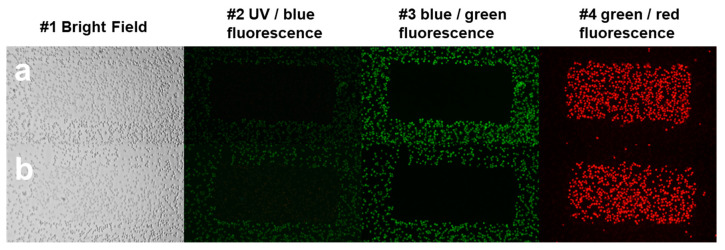
Phototoxicity measurements of HeLa cells labeled with AO or DM. Localized phototoxicity was induced in HeLa cells applying 488-nm laser light illumination. In columns #3 and #4 we can easily distinguish live cells appearing as green (AO or DM staining) from cells targeted to phototoxic death appearing in red (PI staining). The cells were illuminated in 63 cycles in the presence of 2.5 µM AO (**a**, top row images) or DM (**b**, bottom row images) and propidium iodide in both cases with an iCys LSC system. Adherent cells were maintained under physiological conditions during the approximately five-hour procedure. Then, conventional bright-field and epifluorescence microscopic images of the LSC-illuminated area were recorded. Column #1: bright field of view, column #2: UV/blue fluorescence, column #3: blue/green fluorescence and column #4: green/red fluorescence images (excitation light/emission light). A detailed explanation of the experimental setup is described in the caption to Figure 5, and the materials and methods.

After recording the whole process, we recorded wide-field fluorescent images. Figure 4, #3 panel shows how HeLa cells accumulated photosensitizers; the live cells were green at the periphery of panels. The scanned and, consequently, illuminated cells lost their green staining in the middle of the image because of the loss of membrane integrity, and nuclei were stained red at the end of the 63-cycles monitoring process (Figure 4 panels #4). 

The contours of cells can be recognized in the bright field images, both in the middle and periphery of the viewing field (Figure 4 panels #1). The pictures prove that irradiated light energy can be dosed in a localized and quantitative manner. Unilluminated cells provide an inherent negative control.

### 3.7. Real-Time Visualization of the Phototoxicity

**Figure 5:**
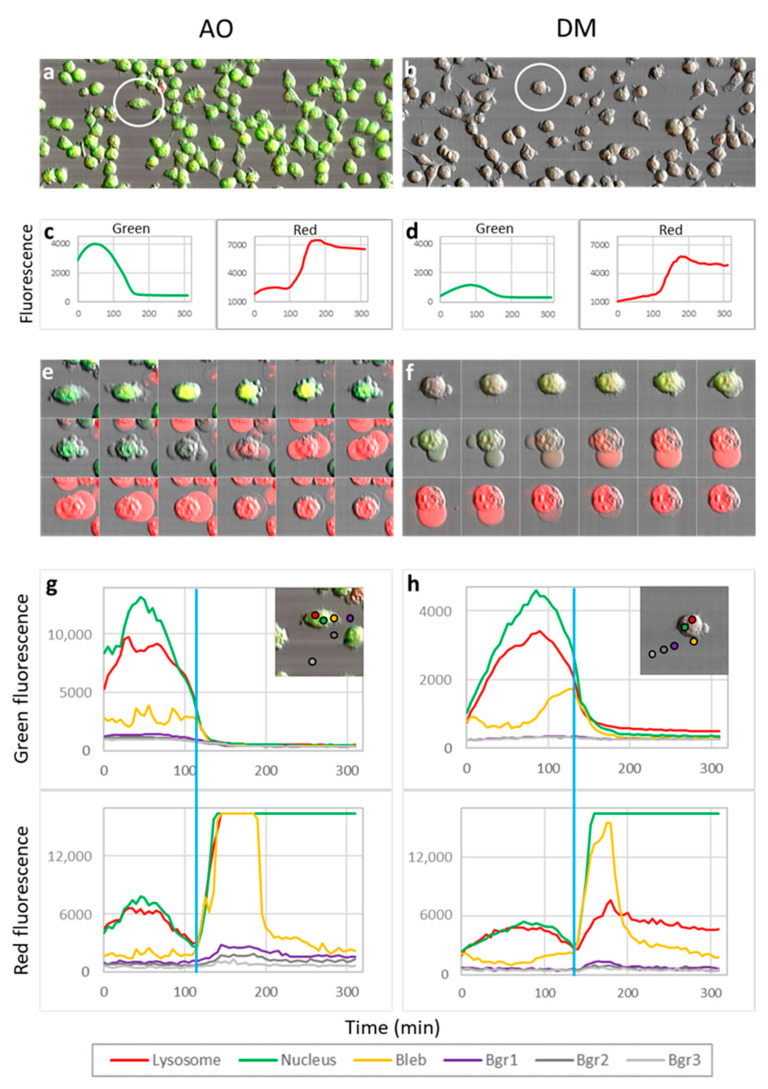
Kinetics of phototoxic cell death of HeLa cells labeled with AO or DM. A shockwave of DNA fragments leaving the nucleus can be recognized with the help of PI staining DNA red (**e**,**f** panels). HeLa cells were pretreated with 2.5 µM AO (left panels: (**a**,**c**,**e**,**g**)), or DM (right panels: (**b**,**d**,**f**,**h**)), and cellular events were monitored in the presence of 7.5 µM propidium iodide. In 63 cycles at 5-min intervals, cells were illuminated by the repetitive scanning of the same area of cells. The corresponding snapshots of the cells were recorded with an iCys LSC imaging cytometry system. Panels (**c**,**d**) show overall kinetics of the green (ex. 488 nm, em. 530/20 nm, (**c**) on the left: AO and (**d**) on the right: DM) and red (ex. 561 nm, em. 580/20 nm) left, PI in the presence of AO and right, PI in the presence of DM) fluorescence in all cells. Panels (**e**,**f**) show 1 representative cell (both for AO and DM encircled by white circles in panels (**a**,**b**)) in 18 subsequent selected frames to emphasize the changes of single-cell events (each image is 75·75 µm^2^). Changes in red PI fluorescence within cellular blebs in the middle row clearly shows the wave of the DNA fragments generated by the photosensitizer mediated 488-nm laser light illumination. Panels (**g**,**h**) quantify the kinetics of photosensitizer loading (green fluorescence in the upper panels) and the generation of DNA fragments (red fluorescence in the lower panels) in color-coded ROIs within and the vicinity of single cells (Lysosome—green, Nucleus—red, Bleb—yellow, and Bgr1—purple, Bgr2—dark grey, Bgr3—light grey) (background fluorescence at three different locations in proportionally increasing distances from the selected cells). The blue vertical line in both the (**g**,**h**) panels mark the inflection points of the OA and DM fluorescence. One representative data set is displayed in this figure from five similar experiments.

Next, we created video films of the images taken in 5-min cycles (Video S1 for AO and Video S2 for DM in the supplementary materials). We could see the accumulation of photosensitizers into the cells, where AO, which has higher fluorescence quantum efficiency than DM, stained cells brighter. The lower quantum efficiency of DM was beneficial for the better discrimination of the appearance of PI within the cells. In the beginning, most of the cells were alive, and just very few dead cells were stained red. First, confluent cells attached to the bottom of the ibidi chamber lose their membrane connections, pseudopodia, and microvilli. Then, cells shrink, membrane fragments are lost, and membrane blebbing is continuously increased. Initially, blebs were small and numerous, then became fewer and grew larger (Figure 5a,b,e,f). Finally, some blebs expanded to cell size, and abruptly, most of the membranous structures retracted to the nuclei’s surface. Only very few blebs remained stable. Blebs at the beginning were transparent, then greenish. Suddenly greenish blebs turned reddish, while nuclei and cytoplasm also switched color. Single-cell changes developed in synchrony with small temporal dispersion; consequently, illumination ignited dye-sensitized cellular changes.

### 3.8. AO Phototoxicity Kinetics Is Faster in Fractionated 488-nm Laser Illumination

In Figure 5, we show the results of the quantitative analysis of the video recordings. Magnified images of one selected cell for both dyes (Figure 5a,b) emphasize the cellular changes developed in all cells (Figure 5e,f). Here, we can better resolve the green–red color transmission, i.e., the loss of photosensitizer from the cell and the augmentation of the DNA signal stained with PI within the cells’ interior. Figure 5e,f shows that the DNA signal intensifies in both the cytoplasm within the blebs’ interior and the nucleus. In physiologic conditions, the chromatin would remain in the nucleus, unless for some reason, the nuclear membrane is lost or the nuclear DNA fragments to the size that they could be freely released from the nuclei. It was evident that large macromolecules are retained in the nucleus since, in the end, red nuclear remnants stained with the highest intensity in dead cells within the whole image sequence.

Nevertheless, vast amounts of reddish DNA are lost from cells, probably as small-sized DNA fragments and these fragments could penetrate damaged nuclear and cytoplasmic membranes fenestrated by light illumination. We wished to quantify DNA lost from cells using fluorescence images. However, there is a big obstacle, which makes this aim impossible. Propidium iodide does not stain intact nuclei well because of the tight supercoiled nature of the DNA in an undamaged nucleus. Similarly, this may be the reason why AO does not stain DNA in live cells well. Intact DNA needs to be relaxed to allow free penetration of dyes, and this relaxation can be achieved either by applying an unwinding compound or by the accumulation of a certain number of DNA nicks [24]. These processes prevent the exact quantitation of fluorescence images of PI-stained DNA in cells monitored continuously under physiologic conditions. Nevertheless, we can quantitate the relative dye content of the images of green and red colors. Figure 5c,d shows the initial accumulation and subsequent loss of photosensitizers integrated for all cells in the pictures. These panels also display the intensification of the DNA staining in the nuclei. These events, i.e., the loss of AO and DM and increasing DNA staining by PI, clearly follow each other in time with a tiny overlap. It can be noticed that AO-driven changes precede the alterations triggered by DM. Cells in every cycle were illuminated with 0.93 J/cm^2^ fluence during one cycle of 2.9-s-long illumination. For the cell killing, 28 cycles were required for DM and 24 cycles for AO (according to the blue vertical lines in Figure 5g,h), and these numbers of exposure cycles correspond to 26.04 and 22.32 J/cm^2^. This observation contrasts with the LED illumination result, where DM was more deadly for cells. The difference between LSC-laser and LED-light illumination was the fractionated versus continuous illumination, in addition to the difference between the 488 and 470 nm excitation wavelengths. After every irradiation period with LSC, cells are left in the dark for approximately five minutes between the cycling 488 nm laser illuminations allowing the cells to regenerate to a certain level after every radiation cycle. The reversal of killing potency of the two dyes in fractionated versus the continuous illumination hints that their primary cellular targets are different.

### 3.9. DNA Fragments Leave Nuclei in a Shockwave-Like Manner

Focusing on the green–red color switching in single-cell frames of the video already suggested that DNA released from nuclei could be visualized by membrane-impermeable DNA dyes, such as PI (Figure 5e,f). We evaluated the time sequences of pixel intensities at different locations around and within the cells (see inserted cellular images at the upper right corners of Figure 5g,h panels). The sites, which were selected as regions of interest (ROI) were color-coded. Changes in the fluorescence intensity of AO were more abrupt than that of DM, and the green–red switching started earlier than with DM. The recognition of these sudden changes is aided by the blue vertical line set to the minimal value of the nuclear red fluorescence. The blue line also coincides with the green inflection point, which is the time reference point for the kinetic analysis. The bottom panels demonstrate the delayed rise of the red background fluorescence compared to the red fluorescence in the nucleus and blebs of the DNA wave. The initially low background fluorescence (purple curve in the bottom of the g panel) jumps for a short time (DNA wave). It then remains higher than the initial baseline value (denoting the DNA released from the cells). The brightest PI signals start with the permeabilization of the plasma membrane. At the same time, the drop of green fluorescence intensity can be observed from the inflection point, which coincides with the initiation of the PI signal increasing enormously at the end, mirroring the PI flood of the nuclear DNA relaxed.

Several additional exciting points can be noticed by analyzing these quantitative data. Firstly, the green-red overlapping period takes a couple of cycles of scanning indicated herein minutes (unit of time on the abscissa). It means that both photosensitizer-loss and PI-increase are retarded slightly within the cytoplasm, whatever mechanism is responsible for it. It cannot be simple dye diffusion because concentration differences would be equilibrated within seconds in these intracellular distances. Therefore, the most probable explanation is that these events reflect some equilibration steps of partially active remnants of living signs of agonizing cells struggling with acute DNA and membrane damages. These equilibrating steps can be, e.g., binding to cytoplasmic or membrane proteins or transmembrane transport of dyes into vesicles and the release from their confinements. Secondly, AO kinetics indicate more pronounced DNA damage because of the faster and more massive PI changes. Both tangents and amplitudes of PI curves for AO show more extensive changes than for DM. Indeed, the extent of fluorescence changes cannot be compared, in terms of DNA contents, since quantum yields for AO and DM are pretty distinct. In the case of AO, all the nuclear, lysosomal, and bleb red signals are saturated quickly, unlike the DM, where only the nuclear signal tops. Thirdly, a shockwave-like abrupt release of DNA can be recognized from the quantitative data of both dyes. In intact cells, there is no nuclear DNA in blebs. However, here, blebs are filled transiently with the strong DNA signal stained by PI. It suggests that AO causes more pronounced DNA damage and produces smaller and more relaxed DNA fragments. Consequently, it makes a brighter PI signal than DM. In the case of AO, this saturated intra-bleb DNA signal (the yellow curve in the lower panel of Figure 5g) drops almost to the background level of signals, clarifying the transient nature of the DNA wave both in space and time. DNA release from cells may happen in conditions when DNA damage occurs, e.g., in the case of apoptotic conditions, or might be happening even locally, e.g., in the case of local caspase activation [25]. Similar systems could be appropriate models for evaluating the DNA release in photodamage or some closely related natural situations. The abrupt DNA damage, which leads to extracellular release and the spreading of DNA fragments to the environment, might be enhanced by the phenomenon observed by Pierzynska-Mach with her colleagues. They observed that the AO-filled acidic lysosomes migrate into nuclear membrane invaginations deep into the nucleus during programmed cell death [17].

### 3.10. Histone H2A Is Also Lost from Nuclei in Photodamage

**Figure 6:**
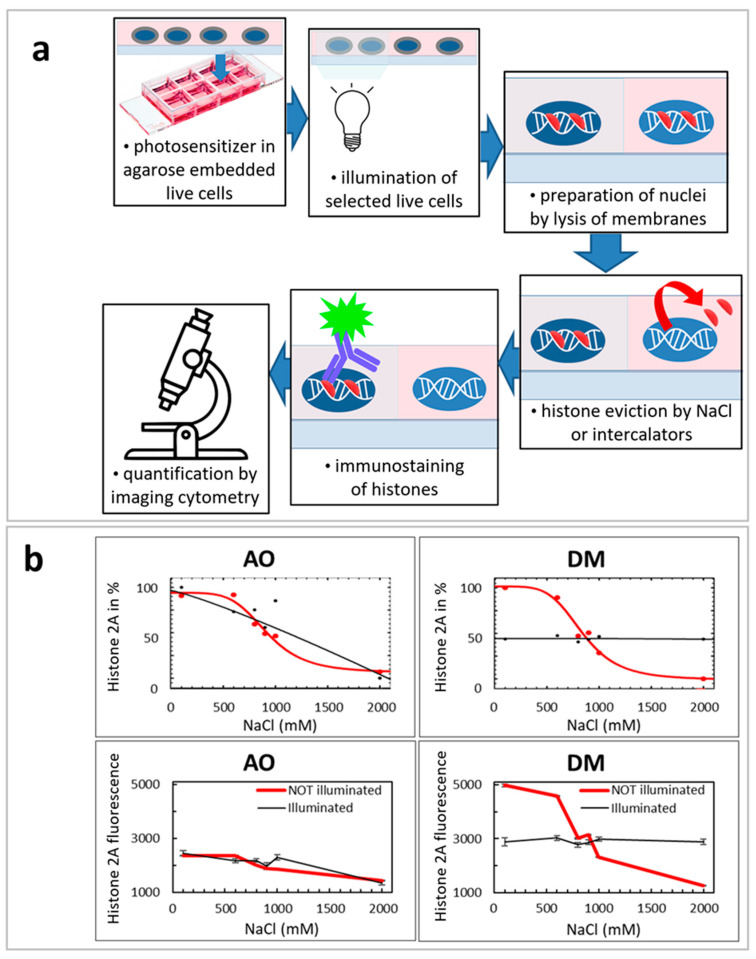
Loss of histone H2A and photofixation in the chromatin. AO evicted more histone H2A from nuclei as a result of the light-induced nuclear DNA fragmentation. DM crosslinked more histone H2A to the chromatin in the illuminated HeLa cells. (**a**) Scheme of the modified QUINESIn method to detect chromatin integrity [24]. Histones are released from nuclei by applying high concentrations of salts unless the photo-treatment crosslinked proteins covalently to the DNA or other proteins. Anti-histone H2A antibodies were used to detect the presence of specific histones. (**b**) The amount of histone H2A was plotted versus the salt concentration. In the upper panels, the relative amount of histone H2A is given in percentages normalized to the no salt condition (100%). In the lower panels, raw data of the histone fluorescence are plotted on the ordinate in arbitrary units (mean ± SEM). The left panels show AO, while the right panels show DM sensitization. All black lines display the light treatment, while red curves label the absence of illumination. In this figure, we demonstrated one representative data set from three experiments.

We wanted to study histone proteins during the photodamage when DNA is lost from the nuclei. We have recently developed a fancy technique called QUINESIn, which measures DNA superhelicity, and quantitates histones and DNA simultaneously in single cells [24]. From those measurements, we knew that by applying high-salt treatment or using appropriate intercalator dyes, histone H2A proteins are better evicted from nuclei than other histones. We utilized the method with slight modification (Figure 6a), i.e., cells were irradiated by the fractionated blue-laser light illumination. Briefly, at the beginning of the experiment, cells stained with photosensitizers were embedded into agarose gel in ibidi slides, such as in single-cell gel electrophoresis. Then cells were illuminated in the gel, maintaining physiologic conditions, in the iCys imaging system. Afterward, cell membranes were lysed in neutral conditions, still in the agarose. Then histones were eluted from the chromatin by applying NaCl solutions with increasing concentrations. Finally, histones remaining in nuclei were stained by fluorescently labeled antibodies, and cells in the ibidi chamber embedded into agarose were visualized and quantified by laser-scanning cytometry. In different ibidi wells, increasing concentrations of NaCl solutions were used to dislodge histones in proportion. The technique results in sigmoid histone eviction curves in normal chromatins (Figure 6b, red curves of the upper panels). In Figure 6b, when unilluminated HeLa cells were treated with AO or DM, histone H2A proteins were released by increasing salt concentrations in a sigmoid pattern (red curves on the left and right upper plots of Figure 6b). When cells were illuminated, we observed that the standard sigmoid shape of the salt-induced histone eviction had changed. A straight line for AO treated cells gave the best fit for data points (Figure 6b, black line in the upper-left panel), and its slope was following the lowering tendency of the sigmoid curve. For the DM illuminated case, data points gave a relatively horizontal line (Figure 6b, black line in the upper-right panel), indicating the photo-crosslinking of dye to the chromatin. In the lower panels of Figure 6, we displayed raw fluorescence data of the histone eviction experiments in arbitrary units. Without illumination, AO treatment evicted more H2A than DM (Figure 6b, compare the beginning of red curves in bottom panels). AO is probably a better DNA intercalator than DM; hence, AO has already evicted half of the histone H2A before the salt treatment (compare red curves on the left and right bottom plots) [24]. The measured changes indicated that histone-crosslinking to the chromatin, DNA fragmentation, loss of DNA from nuclei, and a subsequent histone loss of nuclei occurred during both the AO and DM sensitized photodynamic treatment, although at a different level. First, histone eviction by the DNA intercalating AO is more pronounced (compare red and black curves in the left side plots of Figure 6b) since AO enters cells and nuclei more efficiently than DM. Second, the more substantial histone loss, in the case of AO, is a consequence of the combined effect of the more severe DNA fragmentation with subsequent loss of DNA, the histone eviction by intercalating DNA dyes, and the crosslinking proteins to DNA by the same dye (see the difference in the black illuminated bottom-panel curves). Third, DM crosslinks H2A to DNA better and fragments DNA less than AO (compare black and red lines in the bottom-right panel at high salts).

Summarizing the conclusion derived from the QUINESIn experiments, AO damages chromatin more significantly than DM in fractionated illumination.

### 3.11. Both AO and DM Dyes Cause Lysosomal Permeabilization Induced by Light Illumination

**Figure 7:**
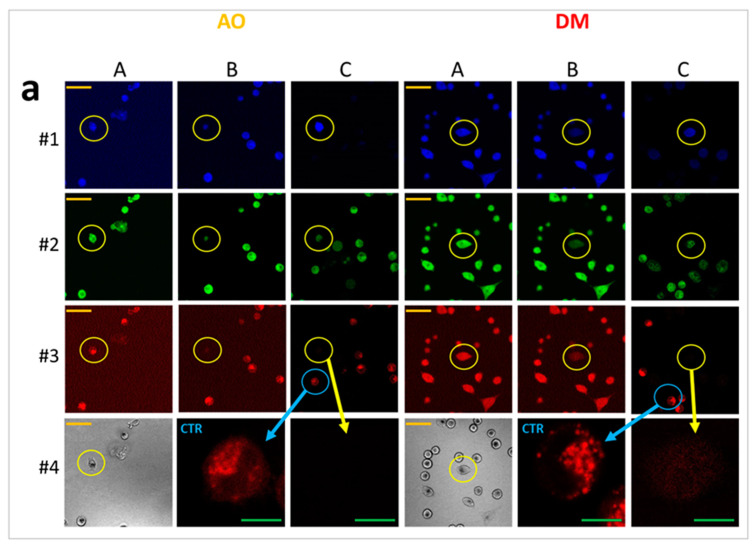

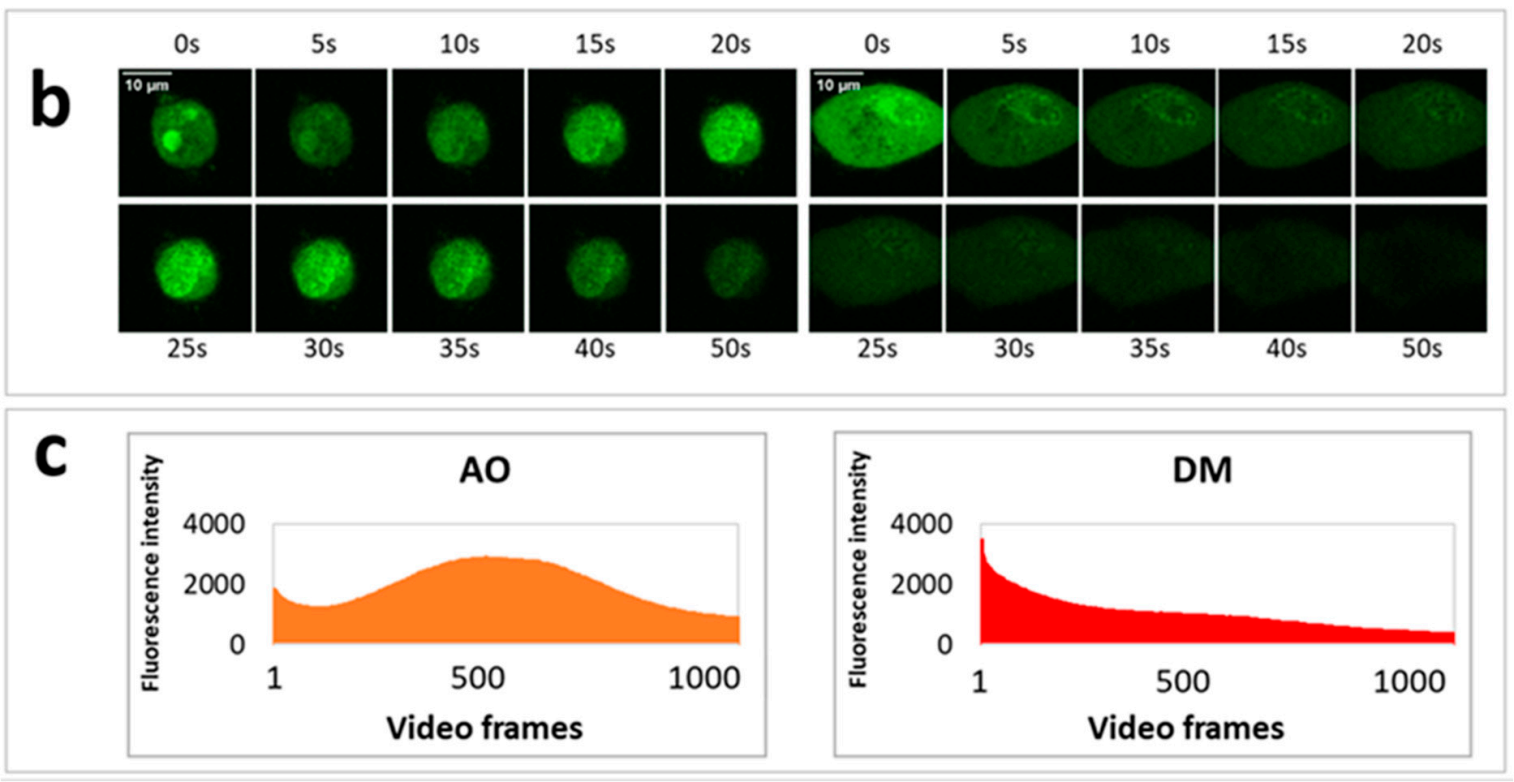
Lysosomal localization of AO and DM in fluorescently-labeled HeLa cells. Both AO and DM dyes cause lysosomal permeabilization for light illumination. To monitor the changes in the integrity of lysosomes, LysoOrange dye was applied. The left side panels show the results of AO treatment, while the right-side panels are the DM results. Cells were pretreated with 2.5 µM concentration of AO or DM for 1 h. Then, the images of the cells were recorded continuously (at 1000 subsequent time points) by sequential laser illumination at 405, 488, and 543 nm. Cells were then stained with LysoOrange for 15 min and re-imaged (**a**), row #4, images B and C). During the experiment, cells were kept at 37 °C. Cell images were taken through various emission filters: 460/20 nm ((**a**), row #1, targeting DM), 530/20 nm ((**a**) row #2, targeting AO), 580/20 nm ((**a**), row #3, targeting LysoOrange). Images in column “A” (rows #1–3) were recorded before the continuous illumination, while in column “B” (rows #1–3), images were taken after the continuous illumination. Images of column “C” (rows #1–3) were taken after the LysoOrange labeling of cells. The “A” image in row #4 of (**a**) are bright-field images of cells that are exposed to the mentioned continuous illumination. These cells are marked with yellow circles. The blue circles show control cells that were not illuminated. In the gallery of (**b**), we see cells at 530/20 nm emission as a function of time when illuminated with the 488-nm laser light. The time is given in seconds in the images. AO images, as a function of time, show a more robust signal initially localized to lysosomes. The sign shows an increase in the transient intensity of nuclei and later fades evenly. In the case of DM, a slower fading of the lysosomes can be observed. (**c**) Shows the integrated intensity of several cells as a function of time. There is an apparent transient increase in the intensity of cells, which is more pronounced for the AO treatment. At the same time, it is hardly seen with DM, but it still exists. At the level of individual cells, this transient increase in the intensity appears in cytoplasmic flashes at the 530/20 nm emission for both dyes. The yellow bar corresponds to 50 µm and the green bars to 10 µm. These experiments were repeated two more times, producing similar results.

It is known that AO also accumulates in live lysosomes and shows red fluorescence because of dimer formation in high concentrations. In blue illumination, AO also damages lysosomes with a spectacular flashing phenomenon [17]. We wondered whether vesicles labeled by DM and demonstrating fluorescence by 405-nm excitation are lysosomes and whether DM damages lysosomes, such as AO. We set up an experiment to visualize AO, DM, and LysoOrange simultaneously while maintaining the same spatially and timely-controlled illumination for cells. Figure 7 shows that both AO- and DM-stained lysosomes and both dyes caused photodamage. Before applying the controlled irradiation, all three fluorophores visualized alike vesicular structures within the cells (Figure 7a, columns A). After localized illumination, the fluorescence of AO and DM dyes disappeared at the tracked location of cells (Figure 7a, columns B), and LysoOrange could not visualize lysosomes within AO or DM-labeled cells (Figure 7a columns C). However, lysosomes in unilluminated but PS-containing cells were stained with LysoOrange, indicating that lysosomes remained intact without light. At the same time, the light caused lysosomal damage in both AO and DM cases. The spectacular flashes of how lysosomes are damaged during the photo illumination of AO-stained cells were also noticeable in DM-stained cells. Although, in DM-stained cells, the phenomenon was less pronounced because of the lower quantum efficiency of the dye (Figure 7b,c). In this method, the onset of the photodestruction is proportional to the membrane sensitivity to rupture. To measure the timing of photodestruction makes this technique a suitable cell biology application for lysosomal stability against peroxidation [26,27,28]. The method has not been verified, until now, with other dyes. Since DM shows similar lysosome destruction, this supports the interpretation of the action of AO and confirms the applicability of the method.

### 3.12. Lysosomal Photodamage of DM Treated Cells Is More Significant Than That of the AO Treated Cells

**Figure 8:**
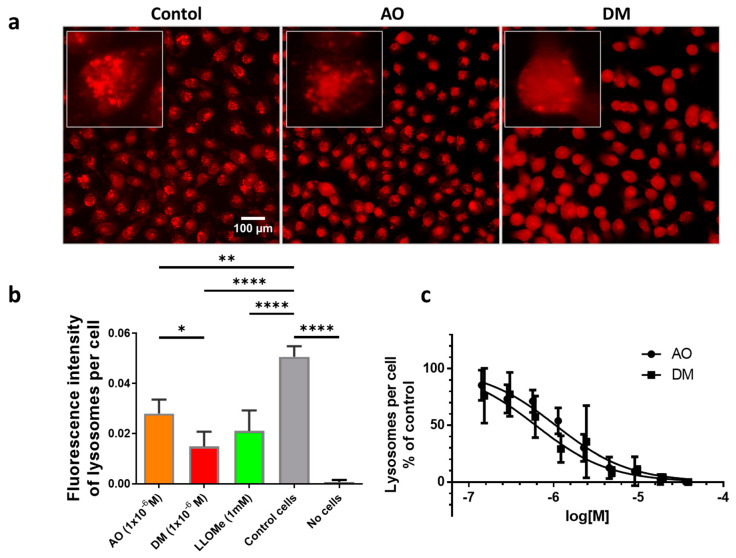
DM causes more significant photodynamic lysosomal damage than AO. The integrity of the lysosomes was examined with LysoOrange dye in HeLa cells. Panel (**a**): Under control conditions (leftmost image), LysoOrange stains many lysosomes around the nucleus with high intensity and contrast, while the cytoplasmic background intensity is low. With blue light illumination after AO and DM pretreatment, fewer lysosomes are stained with the LysoOrange. However, DM has a higher cytoplasmic background intensity (rightmost image) than AO (middle image). In each of the overview images having a low magnification, a representative cell of the sample can be seen magnified. The inserts represent 80·80 µm^2^ in size. Panel (**b**): Image analysis was applied to determine the average fluorescence intensity of the LysoOrange dye in single cells. This average is proportional to the lysosome content of cells. During the DM treatment, significantly fewer intact lysosomes remained in the cells than during AO treatment (*p* = 0.02). LLOMe, a known photodynamic lysosomal damaging compound, was used as the positive control. Panel (**c**): The photodynamic dose-response curves of AO and DM were determined by image analysis of the lysosome integrity. The half-effective dose was 1.1 µM for AO, while 0.6 µM for DM. The statistical comparison of fits indicated a significant difference (*p* = 0.0003) between curves; (*: *p* < 0.05, **: *p* < 0.01, ****: *p* < 0.001). One representative data set out of three experiments is displayed. Details of the image analysis can be found in the supplementary materials.

Verifying that DM also induces photodamage to lysosomes, we aimed to quantify the damage level in a concentration-dependent manner. In a 96-well plate, we treated cells with increasing concentrations of AO and DM. Then we illuminated the cells with blue light continuously in a conventional plate reading experiment. Next, cells were stained with LysoOrange, and fluorescence intensities of the lysosomes were re-recorded. The plate reader’s results were not conclusive. Still, we also made epifluorescence microscopic images of the wells by the color camera of our LSC system and evaluated single-cell images with image analysis. Lysosomes were lysed in correlation with the dye concentration, but cells remained intact. That could be the reason for the failure of the conventional plate reading experiments. However, simple cellular images of wells evaluated on a single cell level correctly identified the lysosomal damage status. Cells unstained with photosensitizer contained punctate lysosomes by LysoOrange with low cytoplasmic background (Figure 8a, control). When we applied illumination with photosensitizers, the number of punctate lysosomes decreased. At the same time, the cytoplasmic background fluorescence increased with the increasing photosensitizer concentrations (Figure 8a, AO, and DM). LLOMe, the positive control for lysosomal membrane disruption, gave similar single-cell images (not shown) to AO and DM. We have evaluated the average lysosomal content of the single cells applying fluorescence images of the LysoOrange stained cells. The number of lysosomes in the treated cells was significantly lower than that of the control cells (Figure 8b), and at 1 µM dye concentration DM disrupted significantly more lysosomes than AO (*p* = 0.02). Then, we fitted the dose-response of the lysosomal inactivation in single cells, which showed that DM gave the more pronounced lysosomal damage. The comparison of fits showed a statistically significant difference (*p* = 0.0003) between AO and DM (Figure 8c). These data indicate that DM damages lysosomes more than AO. Thus, the polar isocyanide group localized asymmetrically in the acridine ring could increase the lysosomal targeting of DM. Probably, this outwardly-directed negative charge has increased the capacity of DM to reach lysosomes. It is also remarkable that continuous illumination, compared to the fractionate one, shifted the origin of cell damage. Continuous irradiation increased the tendency of lysosomal disruption, while DNA damage dominated the scene in fractionated light. These trends suggest that lysosomal membrane repair mechanisms might have a higher capacity than DNA damage repair. To prove this idea requires further experimentation.

## 4. Conclusions

In live cells, DM marks lysosomes with intense green fluorescence at 405-nm laser light excitation. There is no detectable RNA or DNA signal in this emission range, but whole-cell outlines are discernible. During continuous LED lamp excitation at 470 nm, DM has a higher phototoxicity than AO; however, there is no significant difference in their toxicity without light irradiation. Comparing the molecular effect of 488-nm laser light illumination quantitatively, AO proved to be a more efficient DNA damaging agent, while DM was a major lysosome membrane disruptor. On the other hand, in the case of fractional irradiation at 488 nm, AO caused cell death earlier, which was indicated by forming cell membrane blebs first, followed by the shockwave-like spreading of the nuclear DNA. These data suggest that repairing lysosome damage in HeLa cells may have a greater capacity than repairing DNA damage. Overall, our work shows that by increasing the lysosome targeting of AO, the photodestruction of lysosomes can be amplified, possibly causing increased immunogenic cell death induction. This strategy could be worth applying because it can enhance the efficacy of tumor therapy.

## Data Availability

Data sharing is not applicable.

## Supplementary Materials

The following are available online at https://www.mdpi.com/article/10.3390/cancers13225652/s1, Figure S1: UV-Vis absorption and fluorescence emission spectra of AO and DM dyes in the presence and absence of HeLa cells in an aqueous medium, Video S1: Video of acridine orange phototoxicity detected with propidium iodide. Video S2. Video of DM (diMICAAc) phototoxicity detected with propidium iodide. Supplementary materials and methods: Detailed description of the image analysis of cellular lysosome content.
