# Supplementary material for: Isocyanide Substitution in Acridine Orange Shifts DNA Damage-Mediated Phototoxicity to Permeabilization of the Lysosomal Membrane in Cancer Cells
Source: Cancers (Basel). 2021 Nov 12;13(22):5652. doi: 10.3390/cancers13225652 (PMC8616321; doi:10.3390/cancers13225652)
Supplement: Supplementary file 1 [file cancers-13-05652-s001.zip › cancers-1429125-SUPPLEMENTARY-MATERIAL.pdf]

cancers

Isocyanide substitution in acridine orange shifts DNA damage-mediated phototoxicity to permeabilization of the lysosomal membrane in cancer cells

Csaba Bankó, Zsolt László Nagy, Miklós Nagy, Gábor György Szemán-Nagy, István Rebenku, László Imre, Attila Tiba, András Hajdu, János Szöllősi, Sándor Kéki, and Zsolt Bacso

Supplementary Materials

Supplementary figure

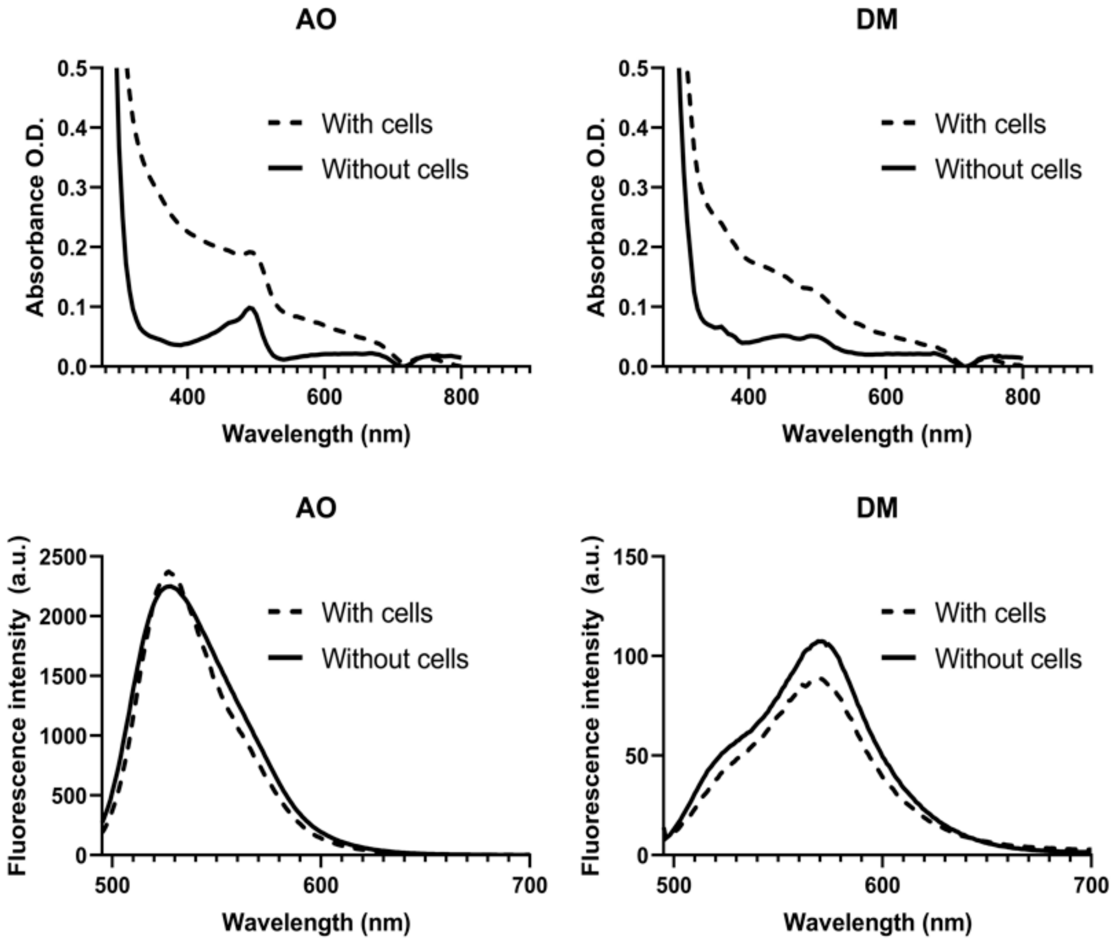

Figure S1. UV-Vis absorption and fluorescence emission spectra of AO and DM dyes in the presence and absence of HeLa cells in an aqueous medium. The two dyes have similar absorption spectra, but the molar absorbance of AO at 480 nm is twice as much as that of DM. At 480 nm excitation, the AO fluorescence intensity at 530 nm was more than twenty times higher than that of DM, while the emission spectrum of DM is red-shifted and occurs at 575 nm. The addition of cell suspension increases the absorbance of the dye solutions due to the turbidity of cell suspension (106 cells/ml). In the presence of the cells, the fluorescence spectra of AO and DM change only slightly.

Supplementary videos

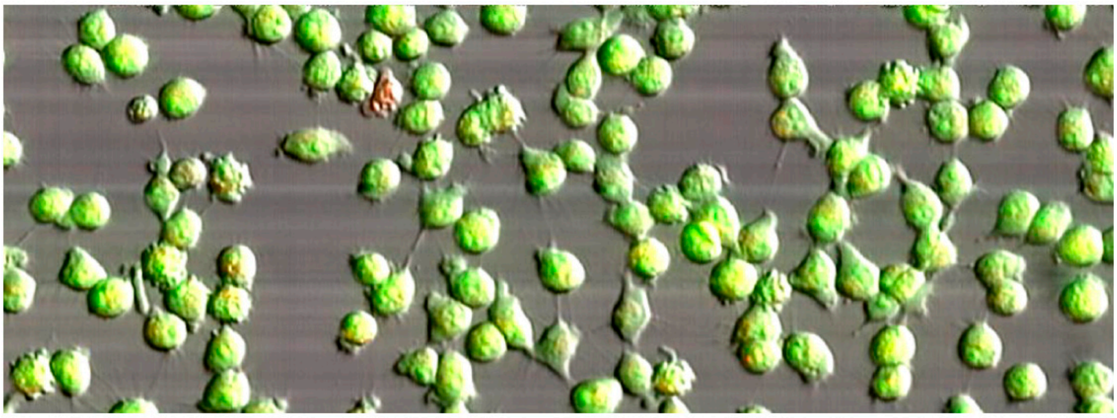

Video S1. Video of acridine orange phototoxicity detected with propidium iodide.

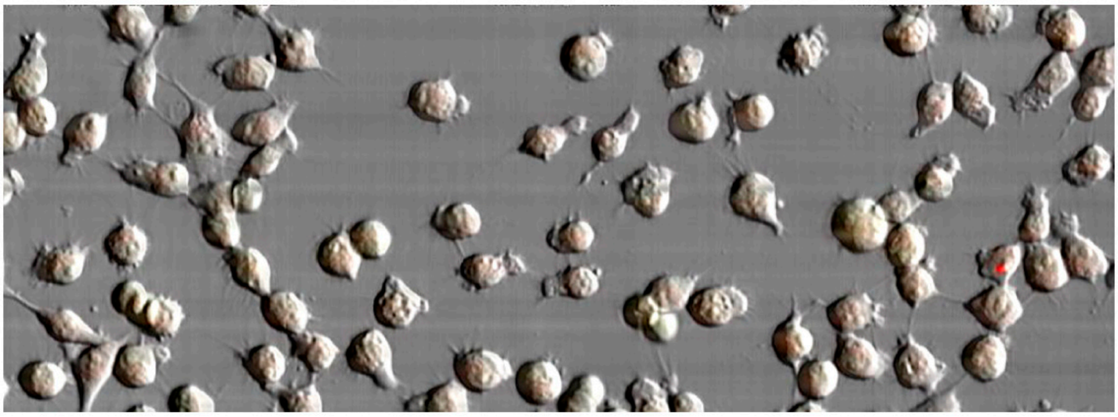

Video S2. Video of DM (diMICAAc) phototoxicity detected with propidium iodide.

Supplementary material and methods

Detailed description of the image analysis of cellular lysosome content

The extraction of the lysosome ratio was performed using digital image processing methods. As a first step, we have used image preprocessing techniques on the input images. Image noise filtering and image sharpening via unsharp masking were applied to perform a more effective segmentation. Unsharp masking is a technique during which sharpening is achieved by subtracting a blurred version of the image from itself [1].

After preprocessing, we have performed the segmentation of the cells. The task is to compose a binary image, where the objects and the background are separated. Consequently, the method separates the foreground from the background. Thresholding regions, where cells overlap and minor impurities of the sample, demand a multistep segmentation method. The first step of the segmentation process is to apply adaptive thresholding [2] to separate the cells from the background. The output of this is algorithm is a binary image containing leaky, imperfect regions making the result slightly noisy and incomplete. To address this problem, we have used morphological operators [1]. These operators are suitable for the correction of segmentation errors, making them appropriate preprocessing tools for the consequent pattern recognition step. Particularly to correct minor noises in the image, erosion is applied to remove points or small patterns in the background that are misinterpreted as belonging to objects.

Moreover, erosion is suitable to separate merged objects. An opening morphological operator is also used for similar purposes to preserve the object size of cells. Some holes may appear along the edges or inside the objects after the segmentation process and application of the erosion and opening operators. In order to fill in these holes, the dilation operator was used. The dimensions of the structuring elements used by the morphological operators were determined uniquely depending on the noise and the sizes of holes. The next step after the extraction of cell bodies was the calculation of the relative amount of lysosomes in cells. The lysosomes inside the cells appear as bright dots; hence, they can be identified as high-intensity pixel regions. We have used a method based on the mean shift algorithm [3] to extract lysosomes, as the method is best suited to find regions with high pixel intensities ('lysosome peaks in the image'). In this process, each pixel value is elevated to the local maximum value closest to it to form the segmented region. The method has a parameter that controls the size of the kernel window. The homogeneity of the cell regions must be taken into account when choosing the parameter. The more homogeneous the image, the smaller the window size has to be used. The calculation of the mean-shift kernel size is required to extract these high-intensity lysosomal spots for the connected cellular regions. Several parameters are considered to reach this aim. Namely, we calculate the mean pixel intensity, but we need to consider each cell's pixel homogeneity, as well, for reality-unbiased expectations. The mean shift threshold level for detecting lysosomes should be applied with different ratios regarding these two values. We have used the homogeneity and entropy first-order statistical descriptors and the second-order statistical descriptors derived from the co-occurrence matrix to measure cell homogeneity [4].

We have found that lysosomal regions obtained by the mean shift algorithms were smaller than authentic regions, so extracted regions were re-grown to reach the real boundary. To determine the boundary of lysosomal regions during region re-growing, we have used gradient maps [4]. The gradient of an image shows the direction and the magnitude of pixel intensity changes at every location. Lysosomal regions begin where the intensity change is large, deriving from the gradient magnitude, and the direction of this change is very steep, calculating from the direction of gradients. To summarize, this method is analogous to the one used in general edge detection.

At the edge of segmented cells, like at cell membranes and regions close to the edge, the intensity values may fluctuate to a large extent, and the segmented region may also be a superset of the actual segmented cell region. To avoid the problems caused by these situations, we do not use pixels close to the cell's edge. For this purpose, we have used the Euclidean distance transform method [5] to create a distance map, which assigns larger values to the pixels closer to the center of the cell while smaller values to the ones closer to the edge. This algorithm can be described as follows: for each pixel in the mask of the image segmentation result, the distance transform assigns a number that is the distance between that pixel and the nearest non-zero pixel of the mask.

We defined the lysosomal ratio of an image as a quotient of the sum of pixels in every lysosomal region and all cells' pixels. This method gives an approximation for the lysosome-cell ratio in an

image. However, it should be mentioned that segmentation does not provide a hundred percent accuracy either for cells or for intracellular lysosomes. Nonetheless, based on our experimental results, the obtained lysosome-cell ratios provide a solid base for comparing the images.

References

1. Gonzalez, R.C.; Woods, R.E. *Digital image processing*. 3rd ed.; Prentice Hall: Upper Saddle River, N.J., 2008; p 954.
2. Bradley, D.; Roth, G. Adapting thresholding using the integral image. *Journal of Graphics Tools* **2007**, *12*, 13-21.
3. Yizong, C. Mean shift, mode seeking, and clustering. *IEEE Transactions on Pattern Analysis and Machine Intelligence* **1995**, *17*, 790-799.
4. Tiba, A.; Harangi, B.; Hajdu, A. In *Efficient texture regularity estimation for second order statistical descriptors*, Proceedings of the 10th International Symposium on Image and Signal Processing and Analysis, 18-20 Sept. 2017, 2017; pp 90-94.
5. Maurer, C.R.; Qi, R.; Raghavan, V. A linear time algorithm for computing exact euclidean distance transforms of binary images in arbitrary dimensions. **2003**, *25*, 265–270.
